# Investigating the Influence of Varied Particle Sizes on the Load-Bearing Properties of Arrester Bed Aggregates

**DOI:** 10.3390/ma17102271

**Published:** 2024-05-11

**Authors:** Pan Liu, Wenju Liu, Peiyi Bai

**Affiliations:** College of Vehicle and Traffic Engineering, Henan University of Science and Technology, Luoyang 471000, China; liuwenju@stu.haust.edu.cn (W.L.); baipeiyi2023@163.com (P.B.)

**Keywords:** discrete element method, arrester bed, load-bearing capability, pebble aggregates, particle size

## Abstract

This study employs the discrete element method to investigate the influence of particle size on the load-bearing characteristics of aggregates, with a specific emphasis on the aggregates used in escape ramp arrester beds. This study utilises the log edge detection algorithm to introduce an innovative approach for modelling irregularly shaped pebbles, integrating their physical properties into a comprehensive discrete element model to enhance the accuracy and applicability of simulations involving such pebbles. Meticulous validation and parameter calibration (friction coefficient: 0.37, maximum RMSE: 3.43) confirm the accuracy of the simulations and facilitate an in-depth examination of the mechanical interactions between aggregate particles at macroscopic and microscopic scales. The findings reveal a significant relationship between the particle size and load-bearing capacity of aggregates. Smaller pebbles, which are more flexible under pressure, can be packed more densely, thereby improving the distribution of vertical forces and increasing the concentration of local stress. This enhancement substantially increases the overall load-bearing capacity of aggregates. These discoveries hold significant implications for engineering practices, particularly in the optimisation of safety for truck escape ramps and in identifying the ideal sizes of pebbles with irregular shapes.

## 1. Introduction

**Figure 1:**
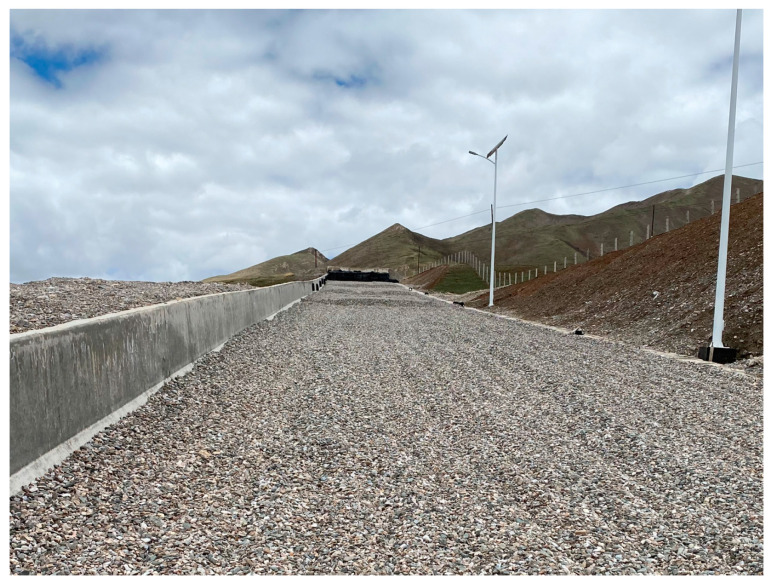
Truck escape ramp.

A truck escape ramp (TER) refers to a designated lane constructed on the outer side of the roadway that is specifically designed for vehicles experiencing braking failures to exit, decelerate, stop, and self-rescue (Figure 1) [1,2,3]. The primary mechanism of TER involves utilising the resistance produced between the truck tyres and the braking surface, typically consisting of pebbles, to effectively decelerate the vehicle [4,5]. However, effective simulation of the driving process of out-of-control vehicles on the escape ramp, especially within the arrester bed, proves challenging with traditional mathematical modelling or road test analysis methods. The core issue arises from difficulty in analysing the load-bearing property of irregularly shaped pebbles randomly distributed within the arrester bed [6], consequently hindering the ability to predict the driving process of tyres within the aggregate. Therefore, the manner in which the load-bearing properties of pebble aggregates with different shapes and sizes is analysed has become a critical issue that must be addressed.

Currently, research into the morphology of particles with irregular shapes has attracted widespread attention [7,8,9,10,11], given their ubiquitous presence across natural and engineered environments, such as rock debris and granular materials. Extensive studies into the morphological construction and physical attributes of these particles have substantially enhanced our comprehension of their unique properties and behaviours.

In the field of particle shape construction methods, Delestre [12] utilised a digital micromirror device for 3D reconstruction of programmable rough particles using interferometric images. Although this technique is effective for reconstructing centrosymmetric and non-centrosymmetric rough particles, its precision is limited for accurately reconstructing particles with complex geometries. Fan [13] devised an innovative method that combines spherical harmonic analysis with a stretching algorithm. This method effectively enhances morphological reconstruction. However, this method requires a substantial augmentation of computational resources and time, especially for analysing large-scale, granular data sets. Trunk [14] developed a method for simulating arbitrarily shaped three-dimensional particles utilising a homogenised lattice Boltzmann approach. This method utilises a discrete representation of particles on a homogeneous grid, diverging from traditional methods that approximate particle shapes using simple analytical equations or their combinations. Liu [15] developed a numerical reconstruction method for depicting irregular 3D particle structures. This approach significantly increases the efficiency of reconstruction and improves the accuracy of shape representation. Nevertheless, the precision of particle shapes must be further improved. Accordingly, imaging techniques, such as cameras and computed tomography (CT) scans, play a crucial role in obtaining 3D shape information on particles. Moreover, strategies that enhance modelling efficiency while ensuring the accuracy of particle shapes must be developed.

In the field of physical characterisation, Mu [16] has analysed particle dynamics within a V-shaped funnel using funnel tests and investigated the shear characteristics of concrete particles. This method effectively simulates and examines the friction and contact properties of particle units. However, the applicability of this method for analysing the load adaptability of pebble layers might be limited. Zhu [17] investigated the influence of particle shape on the mechanical behaviour and fabric evolution of granular materials under complex stress paths using shear tests. These tests play a crucial role in assessing the effects of pebble properties on contact mechanisms. However, considering the typically flat nature of pebble particles, the results of shear tests may exhibit significant variability. Furthermore, this method may not be ideal for assessing the load-bearing capacity of pebble aggregates because of the pressure exerted by a tyre on pebbles. Liu [1] utilised the open-top box compression testing method to calibrate the contact parameters of pebble aggregates. This method effectively simulates the contact characteristics between pebble particles and provides an efficient way to assess the load-bearing capacity of pebble aggregates in arrester beds.

With the continuous advancement in computational capabilities, the discrete element method (DEM) has become increasingly crucial for elucidating the interaction mechanisms among pebble aggregates [18,19,20,21]. The essence of DEM lies in decomposing macroscopic subjects into numerous discrete particle units [22,23,24] and then leveraging Newton’s laws to analyse their interrelations at a microscopic level [25,26]. This analytical approach enables accurate predictions of the dynamic and kinematic behaviour of the investigated entity under varied stress and strain conditions. The application of DEMs extends broadly within the field of highway engineering [27], including the analysis of pavement materials [28,29,30], tyre–soil contact mechanics [31,32,33] and the simulation of trucks travelling on discrete pebble aggregate surfaces [34,35]. Therefore, DEM provides an effective approach for modelling and analysing the load-bearing properties of aggregates with irregular shapes.

## 2. Construction of Pebble Discrete Element Models

### 2.1. DEM Shape Construction Method for Irregularly Shaped Pebbles

**Figure 2:**
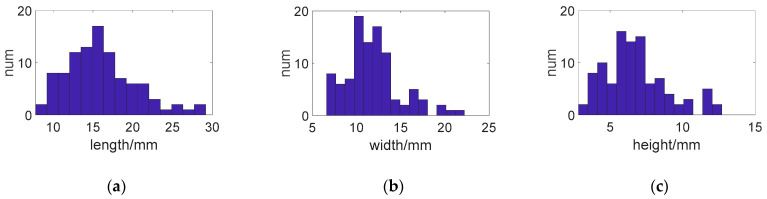
Dimensional distribution of the pebble samples: (**a**) length; (**b**) width; (**c**) height.

The pebble samples used in this study were collected from a TER situated at K209 + 400 on the S308 Provincial Highway in Gansu Province, China. A set of 100 pebble pieces was randomly chosen from the aggregate of the escape ramp’s arrester bed. Figure 2 illustrates the dimensional distribution of these pebble samples.

**Figure 3:**
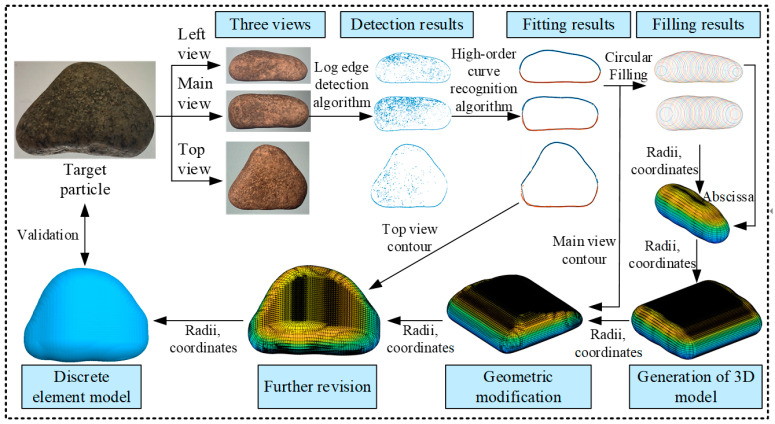
Methodology for shaping a pebble model.

The discrete element method (DEM) was implemented using particle flow code (PFC), and the methodology for constructing the shape of a pebble is illustrated in Figure 3.

The three views of the pebble were photographed, and each of the frontal projections was processed using the log edge detection algorithm [36] to accurately delineate the contours. During the detection phase, noise within the images was eliminated, and the external contour curves were divided into upper and lower sections. The longer sides of each view were extended to a length of 100 units to facilitate subsequent modifications to the particle shape. Additionally, the contour curves were subjected to a higher-order fitting according to Equation (1).

(1) $$z=\sum_{i=1}^{7}a_{i}\cdot x\cdot {\left(x-100\right)}^{i}$$

The contour curves of the pebble, as discerned from the three views, were comprehensively filled using circular units.

The radius, abscissa, and ordinate corresponding to each spherical unit were precisely calculated using Equations (2)–(4).

(2) $$x_{i}=100/n\cdot i(i=1,2,3\ldots n)$$

(3) $$y_{i}=(z_{u}+z_{l})/2$$

(4) $$r_{i}=(z_{u}-z_{l})/2$$

**Figure 4:**
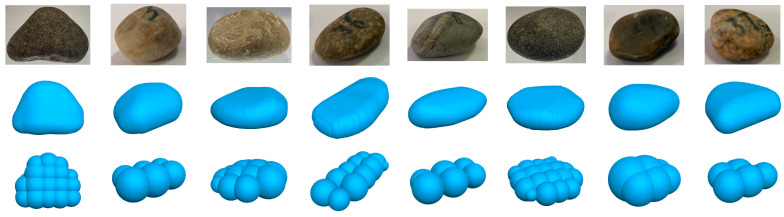
Constructed discrete element model of the pebbles.

A preliminary 3D model was developed using data obtained from the circular elements identified in the left view. In this model, the position coordinates (x, y, z) of each spherical element were specified as (0, x_left, y_left), with x_left and y_left denoting the horizontal and vertical coordinates of the circular elements in the left view, respectively. The model was then replicated and adjusted multiple times based on the x-coordinates (abscissa) of the elements in the primary view. The radii and coordinates of the spherical elements were meticulously recalculated to adhere to the primary view’s contour. This recalibration was similarly executed for the spherical elements according to the top view’s contour. Streamlined pebble discrete element models were established to optimise computational efficiency while preserving the accuracy of the particle shapes (Figure 4).

### 2.2. Calibration of the Parameters for Pebble Discrete Element Models

**Figure 5:**
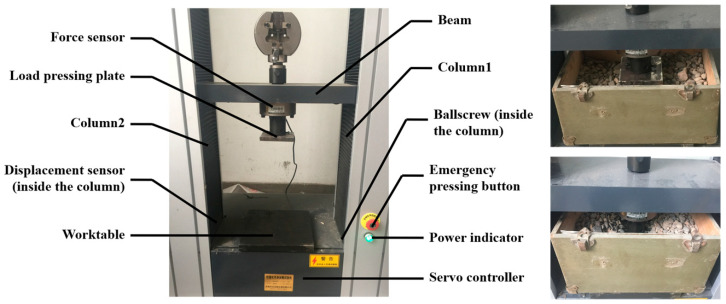
Open-top box compression tests.

Given the primarily rigid characteristics of pebbles, this study utilised the traditional linear contact stiffness model [37] as the foundational contact model in the DEM for pebble aggregates. This contact model is extensively used in research on rigid particles, including rocks and pebbles [38,39]. The key parameters of the pebble DEM included frictional characteristics and were calibrated through open-top box compression tests [15] (Figure 5).

The experimental parameters were as follows: the dimensions of the pressing plate were 0.1 m in length, 0.1 m in width, and 0.01 m in height, while the pebble container measured 0.343 m in length, 0.236 m in width, and 0.164 m in height.

**Figure 6:**
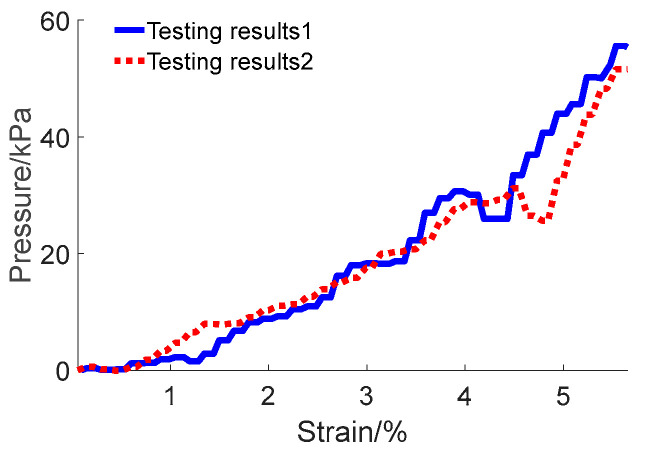
Results of repeated experiments on pebble aggregates in open-top box compression tests.

During the test process, a container was positioned on the worktable. Pebble particles were placed into the container to establish a layer with a thickness of 0.0885 m. Thereafter, the load pressing plate of the test apparatus was lowered into the pebble aggregates at a predetermined speed of 200 mm/min. We thoroughly documented both the strain experienced by the pebble aggregates and the pressure they exerted on the pressing plate. The tests were repeated multiple times to validate the viability of the approach, and the resulting outcomes are depicted in Figure 6.

After the test procedure, a simulation was performed. During the simulation process, the damping coefficient for the pebbles was set at 0.7 [40,41]. The normal stiffness (Kn) and shear stiffness (Ks) values were $4.8\times 10^{6}$ and $2.4\times 10^{7}$ N/m [42]. The density of the pebbles, determined through the drainage method, was 2777 kg/m^3^.

**Figure 7:**
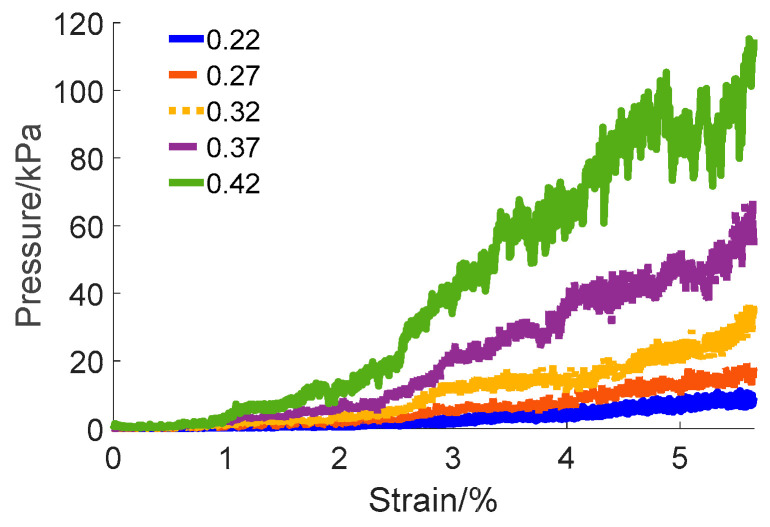
DEM simulation results with varying friction coefficients.

In the initial phase, a 3D model of pebble aggregates with a predefined thickness was constructed inside a container through the rainfall simulation method. A discrete element model was developed for the load pressing plate, which was then assigned a vertical velocity. The plate’s progressive penetration into the pebble was observed throughout the simulation. The strain of pebble aggregates and the pressure they exerted on the pressing plate were meticulously recorded. During the simulation process, the number of particles was set at 70,695, with each trial lasting approximately 7 days. The results demonstrating variations across different friction coefficients are depicted in Figure 7.

It is indicated by the results that the friction coefficient between pebble particles plays a critical role in determining the load-bearing capacity of pebble aggregates. This coefficient influences both the magnitude and the nature of the forces interacting between particles, which are crucial for the transmission and distribution of load and pressure within the aggregate system. A higher friction coefficient increases the resistance to sliding between particles, contributing to a more stable structural formation. Such a structure is more capable of effectively transmitting and dispersing forces under external loads, thereby enhancing the overall stability and load-bearing capacity of the aggregate. In contrast, a lower friction coefficient facilitates easier sliding between particles, potentially leading to quicker deformation or structural collapse under external pressures, consequently diminishing the aggregate’s load-bearing capacity. Based on the results mentioned above, in practical engineering applications concerning the selection of aggregate particles for truck escape ramp arrester beds, it is advisable to choose particles with a rougher surface texture (higher friction coefficient) to enhance the load-bearing capacity of the aggregate.

**Figure 8:**
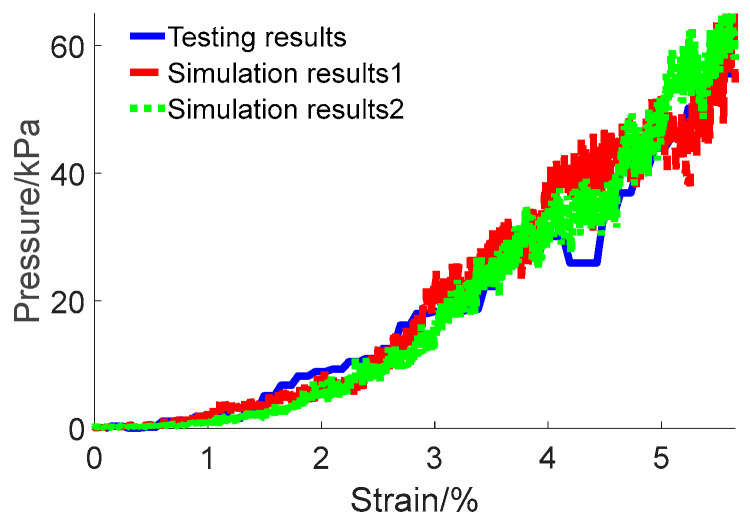
Comparison of open-top box compression test results and simulation outcomes with a set friction coefficient of 0.37.

The friction coefficient of pebble aggregates was determined to be 0.37 based on the results of tests and simulations. These results are consistent with the findings from existing research on similar aggregate materials [43,44], further substantiating the reliability of the calibrated results. Additionally, the simulations were repeated to further validate the results, which are illustrated in Figure 8.

High-order fits were performed separately for the experimental and simulation results. The fitting equation is presented in Equation (5), and the calculated coefficients are shown in Table 1. Compared to the experimental data, the root mean square values for the repeated simulations were 3.37 and 3.43, respectively.

(5) $$y=\sum_{i=1}^{6}a_{i}\cdot x^{6-i}$$

Results from repeated simulations and experiments showed fluctuations, primarily due to the constantly changing states of particle motion, uneven force transmission and distribution, and the dynamic behaviour of force chains within the aggregate. Despite these fluctuations, the overall results demonstrated good consistency, thereby validating the effectiveness of the calibration method.

## 3. Discrete Element Analysis of Mechanical Interactions among Pebble Aggregates

### 3.1. Macroscopic Analysis

**Figure 9:**
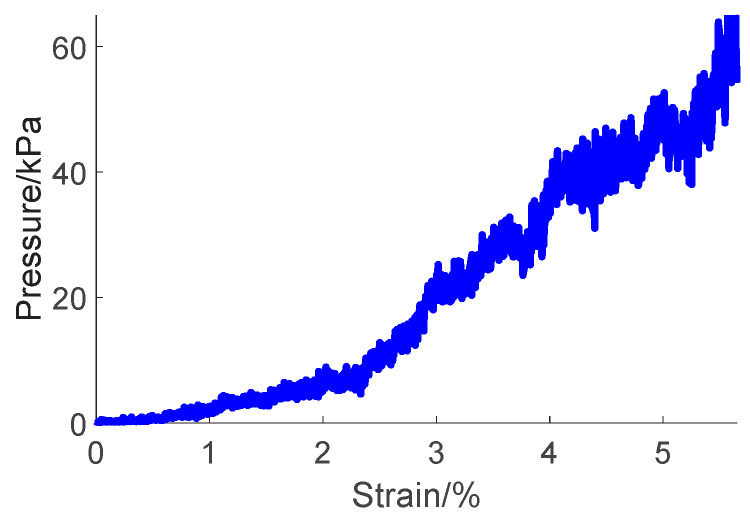
Pressure–strain curve derived from the simulation results.

The pressure–strain curve was derived from the simulation results (Figure 9).

The curve can be divided into two distinct phases: the ‘slow growth phase’ and the ‘rapid growth phase’. In the slow growth phase (strain from 0% to 0.98%), the pressure varies between 0 and 1.96 kPa, indicating a gradual increment. During the transition into the rapid growth phase (strain from 0.98% to 5.65%), the pressure notably surges, peaking at approximately 64.4 kPa.

### 3.2. Microscopic Analysis

A detailed examination of microscopic behaviour was conducted to comprehensively analyse factors influencing the outcomes of the compression results. This task involved scrutinising the motion of pebble particles, analysing the evolving patterns of the force chains interlinking these particles, and assessing the contact forces at the microscopic scale.

(1) Motion of pebble particles

**Figure 10:**
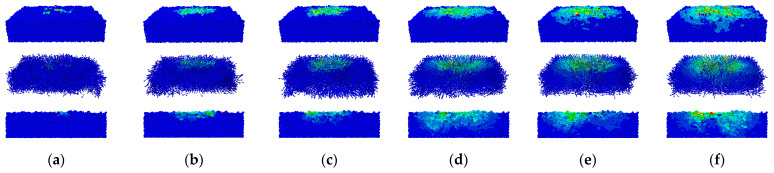
Velocity distribution of the pebble particles. (**a**–**f**) The strain of the pebble aggregate ranges from 0% to 5.65%.

Figure 10 illustrates the velocity of pebble particles and presents a comprehensive analysis from a holistic and longitudinal profile perspective.

The results indicate that pebble particles primarily descend under the pressing plate when subjected to downward pressure. The dispersion pattern of the particles’ response becomes highly pronounced with an increase in the downward displacement of the pressing plate. The analysis of the particle velocity trends at varying depths of compression shows distinct behaviour. When the strain of the pebble aggregate falls within the range of 0% to 1.13%, only the particles in direct contact with the pressing plate are activated. Throughout the deformation process, as the strain increases from 1.13% to 3.39%, particle interactions intensify, impacting a wider area of the aggregates. When the strain of the pebble aggregate falls within the range of 3.39% to 5.65%, the lateral movement of particles adjacent to the aggregate and beneath the pressing plate slows down significantly. These observations indicate that the surface-level particles are influenced by the pressing plate during the initial stages of the compression test. However, the particles beneath the plate collectively move downward as the compression continues, and this phenomenon is driven by the transmission of inter-particle forces. Concurrently, peripheral particles accelerate their movement with the deeper penetration of the pressing plate, while those in the centre, constrained by lateral forces, exhibit minimal positional changes.

(2) Evolutionary patterns of force chains

**Figure 11:**
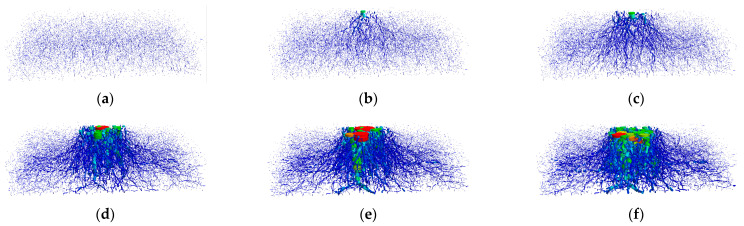
Progression analysis of the contact force chain. (**a**–**f**) The strain of the pebble aggregate ranges from 0% to 5.65%.

The trend of force chain changes throughout the compression process is illustrated in Figure 11.

The force chain displays a root-like distribution pattern. The force chain experiences continuous diffusion with the progressive increase in the displacement of the plate. The central stress is primarily localised in the plate’s lower central region.

(3) Distribution of contact forces

**Figure 12:**
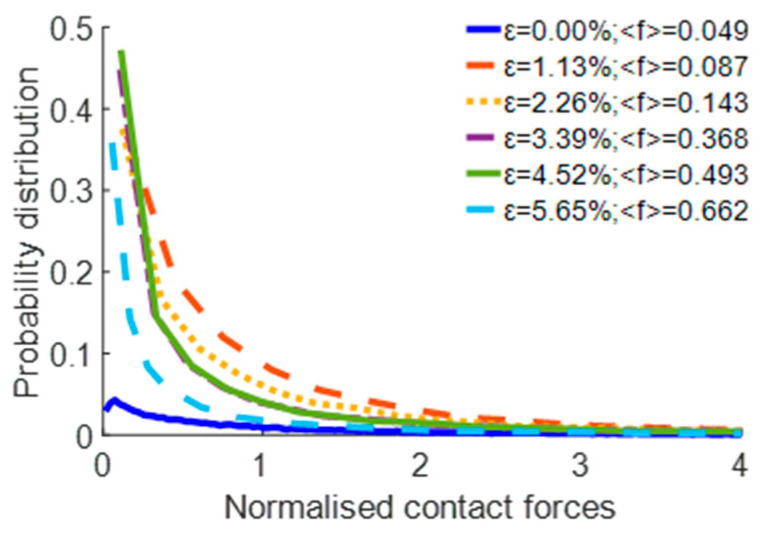
Probabilistic distribution of the normalised contact forces.

All contact forces among the particles were recorded. The probabilistic distribution of normalised contact forces is illustrated in Figure 12.

**Figure 13:**
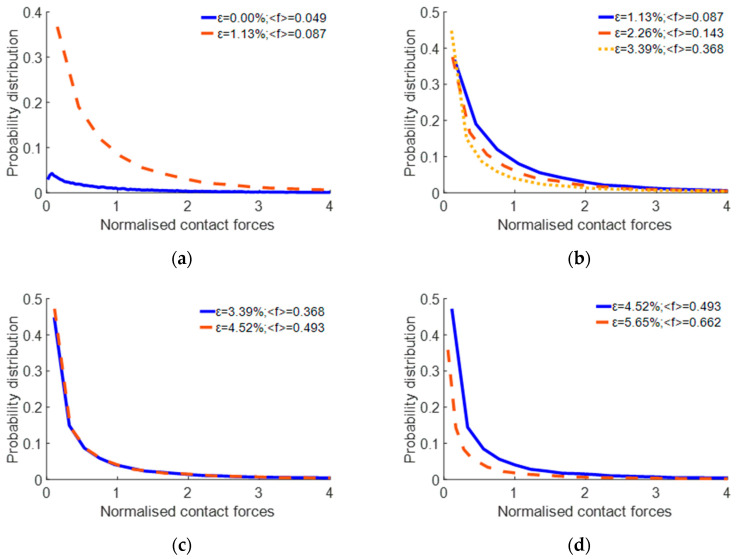
Probability distribution of the normalised contact forces under different strain conditions: (**a**) 0% to 1.13%; (**b**) 1.13% to 3.39%; (**c**) 3.39% to 4.52%; (**d**) 4.52% to 5.65%.

The entire process was divided into four distinct stages to analyse the simulation results at various compression depths (Figure 13).

In Stage 1, spanning the strain range of 0% to 1.13%, a pronounced trend is observed: the overall probability distribution of weaker forces within this interval exhibits a noticeable increase with the deepening of the penetration depth of the pressing plate into the pebble aggregate. This elevation is predominantly due to the emergence of significant forces in specific zones, which in turn increases the average force exerted. Furthermore, the bulk of the original force persists unchanged, resulting in a heightened, weaker force distribution within the 0–1.13% range.

In Stage 2, which corresponds to a strain range of 1.13% to 3.39%, a significant particle dispersion is observed in a downward direction, which can be attributed to the sustained pressure exerted by the plate. This acceleration of the particles’ downward movement facilitates the gradual dispersion of the concentrated stress throughout the sample, thereby decreasing the proportion of weaker forces.

In Stage 3, when the strain falls within the range of 3.39% to 4.52%, the rate of distribution change becomes nearly uniform. The primary reason for this uniformity is the even distribution of stress across the pebbles as the displacement of the pressing plate increases, causing the pressure among particles to proportionally grow. This phenomenon results in minimal changes in the probability of the average force distribution, thereby maintaining a relatively stable state.

In Stage 4, spanning the strain range of 4.52% to 5.65%, a significant shift is observed compared to the previous stage. The speed of the surrounding particles increases as the pressing plate delves deeper, fostering the gradual development of a circulation pattern. This stage is distinguished by a decrease in weaker force distributions, sharply contrasting with the prior stage. The primary reason behind these changes is spatial constraint, which limits the movement of particles internally and markedly enhances the contact forces between the pebbles. Nonetheless, the stress growth among most particles is minimal, resulting in a decreased proportion of weaker forces.

(4) Orientation of contact forces

**Figure 14:**
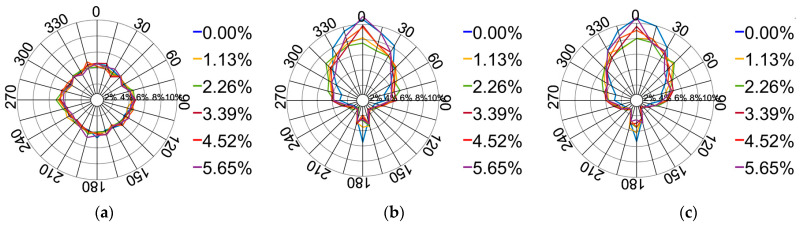
Distribution of the contact force directions: (**a**) XOY plane; (**b**) XOZ plane; (**c**) YOZ plane.

The orientation of the contact forces is depicted in Figure 14. The pressure on the XOY plane displays a symmetrical distribution within a rectangular configuration, closely resembling the shape of the pressing plate. The force distribution on the XOZ plane adopts a leaf-like pattern, primarily concentrated around the longitudinal centre at 0°. Additionally, the force directions on the YOZ plane exhibit a similar distribution pattern. The entire compression process can be categorised into four stages based on variations in the downward displacement of the pressing plate.

**Figure 15:**
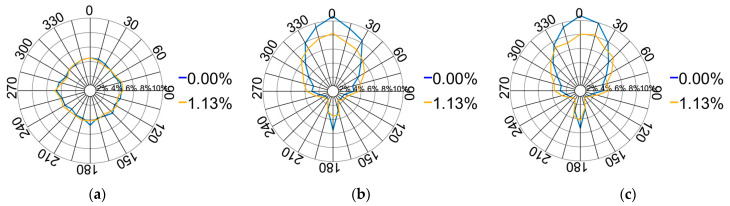
Distribution of the contact force directions from 0% to 1.13%: (**a**) XOY plane; (**b**) XOZ plane; (**c**) YOZ plane.

A comparison of the force directions at two nodal points, with the pebble aggregate strains ranging from 0% to 1.13%, is shown in Figure 15. The variation in the force direction distribution in the XOY plane is less pronounced than that in the XOZ and YOZ planes. The vertical force steadily decreases as the pressing plate continues to descend. This pattern originates from the initial arrangement of aggregate particles formed by the rainfall method, characterised by a predominant vertical force orientation. The particles underneath are compressed downward due to the increasing force applied by the pressing plate, resulting in continuous lateral movement. Consequently, the lateral force increases, while the direction of force distribution remains uniform across various longitudinal profiles.

**Figure 16:**
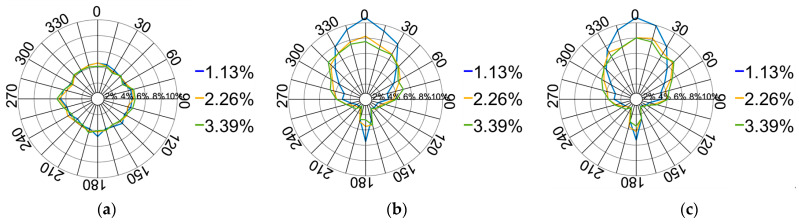
Distribution of the contact force directions from 1.13% to 3.39%: (**a**) XOY plane; (**b**) XOZ plane; (**c**) YOZ plane.

Figure 16 illustrates the contact force directions’ distribution between 1.13% and 3.39%. The vertical force distribution progressively diminishes with a deeper platen displacement. This diminution decelerates when compared to the 0–1.13% range. This deceleration is attributed to the increased depth, causing a rise in the lateral force distribution, even as particles continue to laterally disperse. Nevertheless, spatial constraints reduce this dispersion rate.

**Figure 17:**
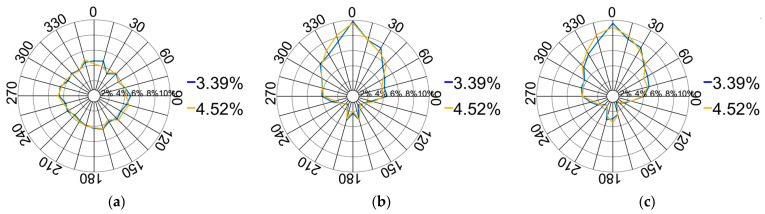
Distribution of the contact force directions from 3.39% to 4.52%: (**a**) XOY plane; (**b**) XOZ plane; (**c**) YOZ plane.

Figure 17 provides a comparison of the force directions corresponding to pebble aggregate strains ranging from 3.39% to 4.52%. The results indicate an exceptionally slight variation in force distribution. This minimal variance is chiefly attributed to the continual lowering of the pressing plate, which progressively compacts the pebble aggregate particles, resulting in a new equilibrium with scarcely any relative positional changes. Thus, the diversity in the direction of applied forces diminishes, guaranteeing consistent force distribution directions across different longitudinal profiles.

**Figure 18:**
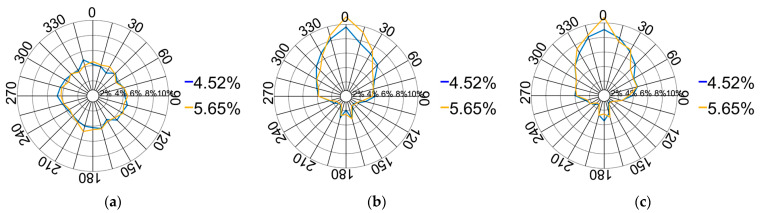
Distribution of contact force directions from 4.52% to 5.65%: (**a**) XOY plane; (**b**) XOZ plane; (**c**) YOZ plane.

Figure 18 illustrates the force direction distribution at pebble aggregate strains ranging from 4.52% to 5.65%. A comparison of nodal forces across different orientations reveals a more pronounced vertical stress distribution. This enhancement is primarily attributed to the accelerated movement of particles around the pressing plate as they penetrate deeper into the aggregate. This acceleration results in a highly scattered distribution of pebble particles, thereby diminishing the distribution of lateral forces.

### 3.3. Integrated Macroscopic and Microscopic Analyses

The entire process can be segmented into four stages. In Stage 1, ranging from 0% to 1.13%, only the loosely packed pebble particles in direct contact with the pressing plate undergo compression. This phenomenon results in the emergence of significant forces in specific zones, subsequently elevating the average contact force. Concurrently, the majority of the initial force remains constant, which intensifies the distribution of weaker forces. The orientation of contact forces is influenced by the initial arrangement of aggregate particles formed through the rainfall method, which is characterised by a predominant vertical force orientation. The particles underneath are compressed downward, resulting in their continuous lateral movement due to the increasing force applied by the pressing plate.

In Stage 2, spanning from 1.13% to 3.39%, the surface particles undergo gradual compression, and interactions among particles intensify, influencing a wider area of the pebble aggregates. The particles beneath the plate collectively undergo a downward movement. This acceleration in the particles’ downward motion facilitates the gradual dispersion of concentrated stress throughout the sample, resulting in a reduced prevalence of weaker forces. During this process, in comparison with the 0–1.13% range, the deceleration is attributed to the increased depth, causing an increase in lateral force distribution, even as particles continue to disperse laterally. Nonetheless, spatial constraints impede this lateral dispersion rate.

In Stage 3, covering the range from 3.39% to 4.52%, the ongoing descent of the pressing plate steadily compacts the pebble aggregate particles, resulting in a proportional increase in pressure among the particles. This phenomenon results in minimal alterations in the probability of the average force distribution. Furthermore, the diversity in the direction of applied forces diminishes due to the minimal relative positional changes among particles, ensuring consistent force distribution directions.

In Stage 4, covering the range from 4.52% to 5.65%, spatial constraints restrict the internal movement of particles and significantly increase the contact forces between pebbles. Nevertheless, the stress growth among most particles is minimal, resulting in a decreased distribution of weaker forces. Concurrently, the accelerated movement of particles around the pressing plate results in a scattered distribution of pebble particles and a reduced distribution of lateral forces.

## 4. Influence of Particle Size on the Load-Bearing Capacity of Pebble Aggregates

### 4.1. Pressure–Strain Analysis

This study investigated the effect of varying particle sizes on the load-bearing capacities of pebble aggregates. The real pebble sizes collected from truck escape lanes were taken as a reference (the dimensional distribution is shown in Figure 2). The pebbles were proportionally enlarged across all dimensions to establish particle sizes for various simulated experimental groups. The enlargement ratios applied were 1.1, 1.3, and 1.5. For clarity in discussion, these particles were designated as small, medium, and large, respectively.

**Figure 19:**
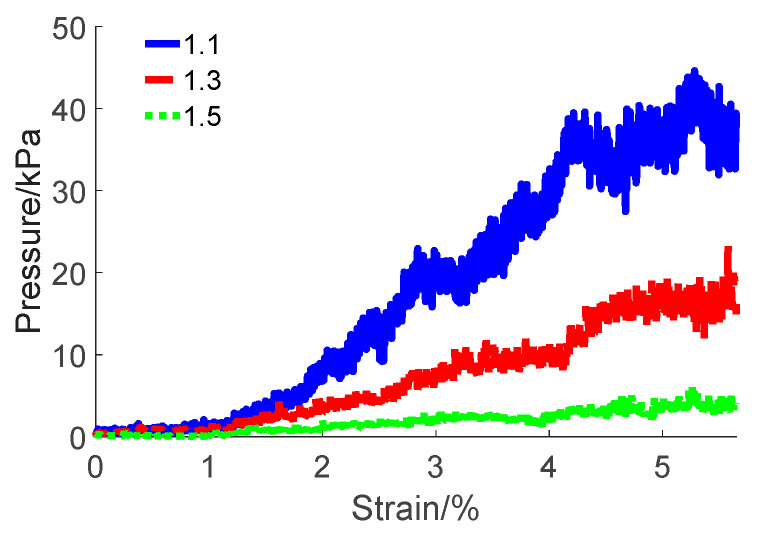
Pressure–strain curve for different pebble size ratios.

The simulation results for the pressure–strain curve of the various particle sizes are illustrated in Figure 19.

The curve exhibits two distinct phases, aligning with the observations depicted in Figure 19. In the slow growth phase, the strain of the pebble aggregate ranges from 0% to 1.13%, while the pressure varies between 0 and 2 kPa. Throughout this phase, the pressure–strain relationship of the particle sizes demonstrates minimal fluctuations. Transitioning into the rapid growth phase, the strain of the pebble aggregate extends from 1.13% to 5.65%. Notably, particle sizes with diameter ratios of 1.1, 1.3, and 1.5 exhibit distinct peaks, reaching approximately 38.80, 17.72, and 3.78 kPa, respectively. These findings underscore a clear correlation between the bearing capacity of the pebble aggregate and the size of its particles. Specifically, within a given depth of the pressing plate, larger particles exert less force on the pressing plate. This observation suggests an intrinsic connection between the bearing capacity of the pebble aggregate and its particle size.

### 4.2. Analysis of the Average Contact Forces

The average tangential, normal, and resultant forces are presented in Table 2.

The table demonstrates a clear trend: the average combined force exerted on the aggregate increases with the increasing depth of the plate. Within this resultant force, the normal force emerges as the predominant component, while the tangential force plays a secondary yet significant role. The normal and tangential forces consistently show an increase in response to the progressive depth of platen penetration. At a strain of 0%, the average contact stress synchronously increases with an increase in particle diameter. The primary reason for this phenomenon is the relationship between particle size, volume, and gravity. In scenarios without platen pressure, larger particles experience heightened gravitational force due to their increased volume. This force results in an increased contact force between larger particles. The inter-particle stress also increases with increasing average particle size as the platen displacement increases. However, the relative difference becomes smaller. When the strain reaches 2.26%, the forces acting on the pressing plate under smaller particle sizes are reversed and further widen the gap as the pressing plate continues to press down.

### 4.3. Investigation of Contact Forces within Pebble Aggregates

**Figure 20:**
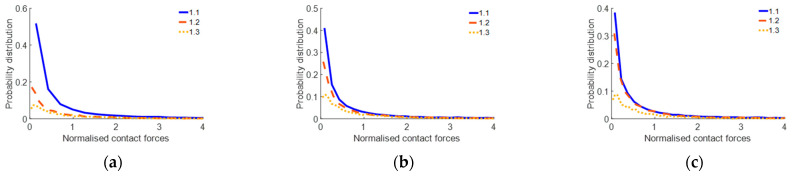
Probabilistic distribution of the normalised contact forces at different pebble size ratios: (**a**) strain of 3.39%; (**b**) strain of 4.52%; (**c**) strain of 5.65%.

**Figure 21:**
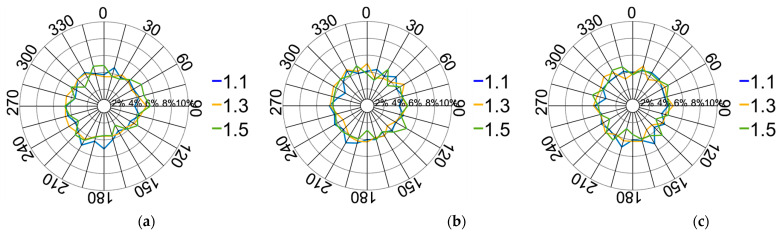
Distribution of the contact force directions on the XOY plane at different pebble size ratios: (**a**) strain of 3.39%; (**b**) strain of 4.52%; (**c**) strain of 5.65%.

**Figure 22:**
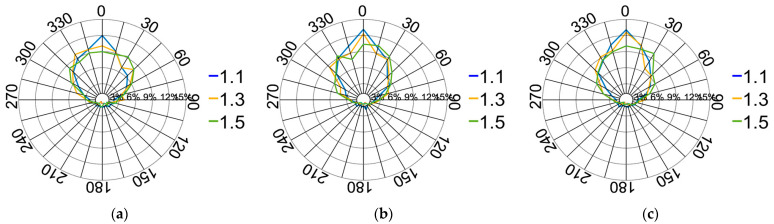
Distribution of the contact force directions on the XOZ plane at different pebble size ratios: (**a**) strain of 3.39%; (**b**) strain of 4.52%; (**c**) strain of 5.65%.

During the initial stage (strains ranging from 0% to 3.39%), the overall force chain framework remains unformed, in accordance with the analysis and explanation provided in Section 3.3 of this work. This study exclusively analysed the state when the force chain framework is formed (strains ranging from 3.39% to 5.65%) to precisely evaluate the load-bearing capacity of the pebble aggregates. The distributions of normalised contact forces and contact force directions on the XOY and XOZ planes for different pebble size ratios are illustrated in Figure 20, Figure 21 and Figure 22.

The results indicate that smaller particles are more likely to readjust their positions under the force, enabling them to densely pack within a given volume. This high-density arrangement results in an increased frequency of mutual contact among particles, resulting in a more concentrated local stress compared with larger particles, thereby increasing the average force. Furthermore, the majority of the original force remains unchanged, contributing to an amplified, weaker force distribution. Larger particles exhibit a relative looseness among them under the same strain. Such dynamic adaptability promotes a more uniform distribution of stress among multiple contact points within the aggregate. This acceleration results in a more scattered distribution of pebble particles, correspondingly reducing the distribution of vertical forces. Additionally, the observed pressure distribution in the XOY plane of the pebble aggregate remains consistent across different particle sizes. Therefore, the XOY pressure distribution is primarily influenced by the size of the compressing plate, with a relatively minor correlation to particle size.

## 5. Conclusions

In this study, the load-bearing capacity of pebble aggregates with varied particle sizes was analysed. (1) A novel construction methodology aligned with irregularly shaped pebble aggregate particles was proposed utilising DEM coupled with the log edge detection algorithm. The key parameters, like the friction coefficient, were calibrated through open-top box compression tests to be 0.37, and the corresponding maximum RMSE was 3.43, thereby validating the effectiveness of the calibration method. An analysis method for the load-bearing capacity of pebble aggregates from the macroscopic and microscopic perspectives was introduced. (2) An investigation was conducted on the influence of particle size on the load-bearing capacity of pebble aggregates. The pressure–strain curves for pebble aggregates of varying particle sizes demonstrated pronounced peaks during compression when the strain reached 5%, with pressures of approximately 38.80 (small pebbles), 17.72 (medium pebbles), and 3.78 (large pebbles) kPa. These results clearly correlate the load-bearing capacity of the pebble aggregates with their particle sizes. Additionally, the statistical analysis of inter-particle contact forces revealed that the squared values of tangential forces constituted approximately 8% of the resultant forces’ squared values, while normal forces accounted for about 92%. The results indicated that in the initial state, larger particles experienced heightened gravitational force due to their increased volume, resulting in an increased contact force between them. Smaller pebble particles were more likely to have their positions readjusted under the force as the pressing plate penetrated deeper, enabling them to be densely packed within a given volume, thereby increasing the distribution of vertical forces. This phenomenon resulted in a more concentrated local stress, which increased the average force and significantly enhanced the load-bearing capacity of the pebble aggregates.

In the future, as computational power and software capabilities improve, along with the availability of comprehensive compression test results, in-depth analyses on the load-bearing effects of large-scale particles will be conducted to develop more representative pressure–strain curves. Additionally, future research will investigate the load-bearing capacity of aggregates under various conditions, including size polydispersion, sieving curves, shape indices, shape characteristics, porosity, and temperature and humidity levels.

## Figures and Tables

**Table 1:** The fitting coefficient of determination.

	a1	a2	a3	a4	a5	a6
Testing results	0.02614	−0.1935	−0.02807	3.908	−2.677	0.6594
Simulation results 1	0.1836	−2.635	13.05	−24.02	18.15	−2.664
Simulation results 2	0.06367	−0.8841	4.241	−5.78	3.305	−0.1568

**Table 2:** Average tangential, normal, and resultant forces.

Average Force	Ratio	0%	1.13%	2.26%	3.39%	4.52%	5.65%
Tangential forces (N)	1.1	0.0119	0.0176	0.0605	0.1140	0.1614	0.1778
	1.3	0.0169	0.0234	0.0479	0.0831	0.0987	0.1367
	1.5	0.0238	0.0267	0.0400	0.0460	0.0610	0.0666
Normal forces (N)	1.1	0.0535	0.0697	0.2115	0.4020	0.5780	0.6259
	1.3	0.0730	0.0904	0.1688	0.2957	0.3529	0.4883
	1.5	0.0949	0.1042	0.1456	0.1613	0.2137	0.2331
Resultant forces (N)	1.1	0.0551	0.0722	0.2210	0.4198	0.6032	0.6538
	1.3	0.0752	0.0938	0.1763	0.3086	0.3684	0.5097
	1.5	0.0983	0.1080	0.1517	0.1685	0.2233	0.2436

## Data Availability

Data are contained within the article.
